# Pink Tooth of Mummery in the Maxillary Left Canine after Fixed Partial Denture (FPD) Preparation

**DOI:** 10.1155/2019/2096120

**Published:** 2019-11-13

**Authors:** Eger V. Korolevsky, Takashi Komabayashi, Denise Foran

**Affiliations:** ^1^Department of Veterans Affairs, New York Harbor Healthcare System, New York City, NY, USA; ^2^University of New England College of Dental Medicine, Portland, ME, USA

## Abstract

Pink tooth of Mummery is typically found after trauma. However, this case report describes an unusual occurrence of pink tooth in a 67-year-old Caucasian male after fixed partial denture (FPD) tooth preparation. Pink tooth in this case may be due to one or more factors: tooth reduction and heat generation during tooth preparation; heat generation during polymerization of provisional material; and hyperocclusion of a provisional FPD. This case highlights the importance of choosing the appropriate materials and techniques to avoid pulpal complications after dental prosthesis work.

## 1. Introduction

A tooth with a pinkish hue is classically described as the pink tooth of Mummery, named after the 19th century anatomist James Howard Mummery. It is speculated that a pink tooth is due to the loss of internal dentin; this loss creates a large pulp space and allows more blood vessels to fill the area, which results in a pinkish hue. A pulpal hemorrhage is defined as the escape of blood from a ruptured vessel, and blood is trapped inside of the pulp chamber, giving off a pink hue. Thus, the pink tooth is usually associated with internal resorption in the coronal area of a tooth [1]. While pink tooth often emerges after a dental trauma or following orthodontic treatment [2], few reports refer to the appearance of pink tooth after a fixed partial denture (FPD), also known as bridge, tooth preparation.

The aim of this case report is to describe an unusual case of a pink tooth of Mummery occurring during fixed partial denture (FPD) tooth preparation and to identify possible causes.

## 2. Case Report

### 2.1. Previous Treatment and Diagnosis

A 67-year-old Caucasian male presented to the Veterans Affairs New York Harbor Medical Center's Manhattan Endodontics Department for evaluation of dental pain on maxillary left canine, tooth #11. Six weeks prior to this evaluation appointment, maxillary teeth had been prepared by a dentist for a fixed partial denture (FPD) spanning from tooth #6 (maxillary right canine) to tooth #11 (maxillary left canine). Teeth #6 and #11 are abutment teeth. Teeth #7 (maxillary right lateral incisor), #8 (maxillary right central incisor), #9 (maxillary left central incisor), and #10 (maxillary left lateral incisor) are pontics of the FPD. The patient's chief complaint four weeks after the initial preparation of teeth #6 and #11 was the lingering sensitivity to cold in the maxillary left quadrant. When the patient presented to the endodontic department six weeks after the teeth were prepared for the FPD, his sensitivity had subsided.

His medical history was significant for sciatica, osteoarthritis with herniated disc, anxiety, and depression. He was taking gabapentin 300 mg tab twice a day for back pain, methocarbamol 750 mg tab three times a day for muscle relaxation, venlafaxine 75 mg tab once a day for depression, sertraline 200 mg tab once a day for anxiety, quetiapine fumarate 400 mg tab twice a day for depression, and diazepam 5 mg tab once a day for anxiety. He had drug allergies to haloperidol and risperidone. His dental history included a full mouth rehabilitation with low caries risk and acceptable oral hygiene.

**Figure 1:**
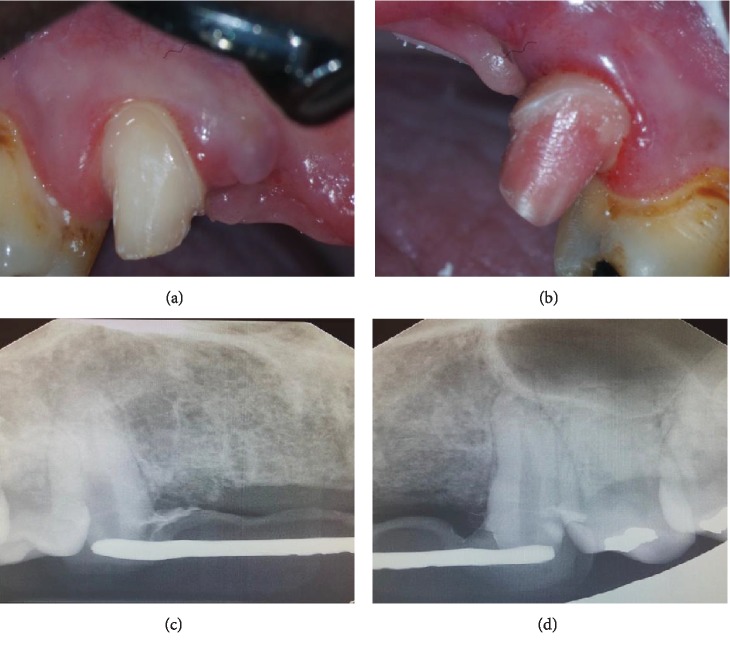
(a) Clinical photograph of tooth #6 after the removal of provisional FPD, normal clinical appearance. (b) Clinical photograph of tooth #11 after the removal of provisional FPD, red hue appearance. (c) Radiograph of tooth #6 before the removal of provisional FPD. (d) Radiograph of tooth #11 before the removal of provisional FPD.

The extraoral exam and perioral soft tissue exams were within normal limits. His temporomandibular joint function was normal without any deviation upon opening or discomfort upon palpation, and the intraoral hard examination revealed no evident abnormalities; the soft tissues had no signs or symptoms of pathology. Clinically, teeth #6-11 were temporized with Luxatemp LC (DMG America, Englewood, NJ, USA) provisional cemented with Durelon (3M, Two Harbors, MN, USA). There was discomfort upon percussion and no tenderness upon palpation (Table 1). His chief complaint was not reproducible with Endo Ice (Coltène Whaledent, Cuyahoga Falls, OH, USA) when tested with the FPD in place. Teeth #6 and #11 responded to cold without lingering sensitivity. The FPD was removed without local anesthesia to further evaluate the teeth. Tooth #11 appeared to have a bright red hemorrhage-like hue whereas tooth #6 appeared normal (Figures 1(a) and 1(b)). A radiographic examination revealed an intact periodontal ligament (PDL) space around tooth #11 with no obvious periapical pathology (Figures 1(c) and 1(d)). Pulpal diagnosis was asymptomatic irreversible pulpitis, and apical diagnosis was symptomatic apical periodontitis. Treatment options were discussed with patient, including no treatment, nonsurgical root canal treatment (NSRCT), or extraction. Considering risks and benefits of all treatment options, an agreement was made to proceed with NSRCT and informed consent was obtained.

### 2.2. Endodontic Treatment

**Figure 2:**
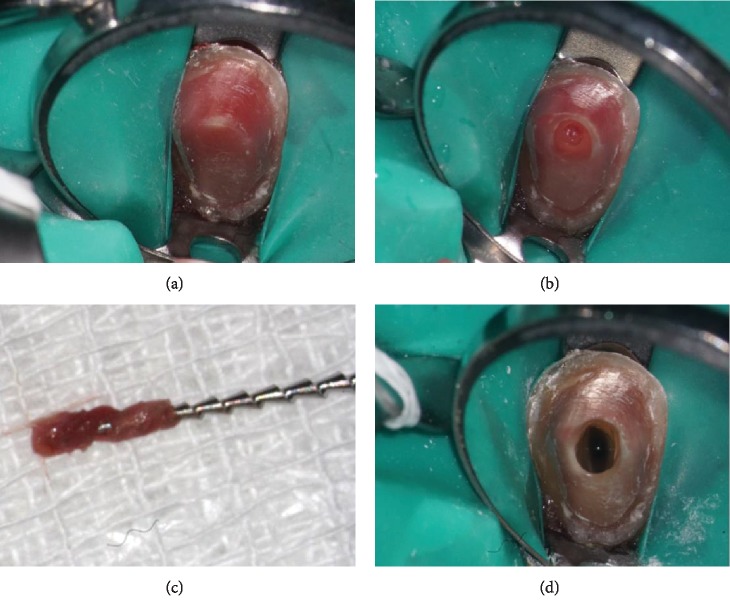
(a) Rubber dam isolation of tooth #11. (b) Initial access preparation showing hemorrhagic pulpal tissue. (c) Extirpated pulpal tissue on hedstrom file. (d) Irrigation with 6% sodium hypochlorite solution in a pulp chamber. Tooth immediately changed color due to oxidation of hemoglobin.

**Figure 3:**
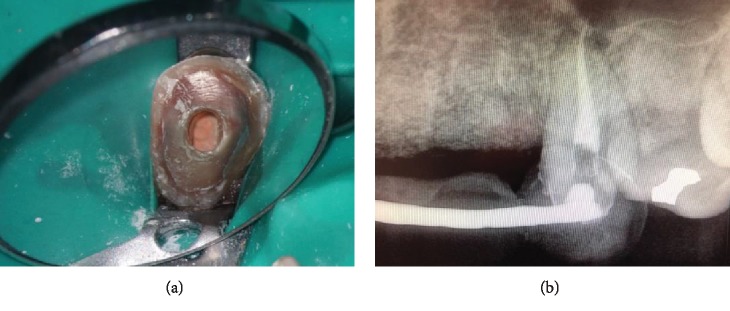
(a) Clinical photograph of the obturated tooth #11 prior to placing a temporary filling and the provisional FPD. (b) Postoperative radiograph of the obturated tooth #11 with the provisional FPD cemented.

The endodontic treatment for this tooth was initiated the day of the evaluation. Patient was anesthetized via maxillary infiltration with 1.8 ml of 2% lidocaine with 0.036 mg of epinephrine. Tooth isolation was achieved with a ligated endodontic clamp and a latex rubber dam (Figure 2(a)). With the use of a dental operating microscope, hemorrhagic vital pulp tissue was found inside the pulp chamber upon access (Figure 2(b)). Although the pulp tissue was still vital, the tissue did not have a healthy appearance (Figure 2(c)). Pulp extirpation was accomplished using hand instrumentation with K-type and Hedstrom files (Dentsply Sirona, Tulsa, OK, USA) and rotary instrumentation using HyFlex EDM files (Coltene, Cuyahoga Falls, OH, USA). Working lengths were determined with the use of an electronic apex locator (Root ZX II, J. Morita, Kyoto, Japan), and measurement radiographs were taken with a size 30K file. Copious irrigation of 6% sodium hypochlorite solution was used during the cleaning and shaping process, followed by a final rinse of 17% ethylenediaminetetraacetic acid (EDTA) to remove the smear layer (Figure 2(d)). Gutta percha 0.04 taper size 25 (Henry Schein, Melville, NY, USA) was selected to obtain an appropriate apical fit and tug back. After taking a master cone radiograph, the canal was dried with medium size paper points (DiaDent, Seoul, Korea) and obturated using a warm vertical condensation technique with a BL-alpha II heating plugger (B&L Biotech USA Inc, Fairfax, VA, USA) in conjunction with Sealapex root canal sealer (SybronEndo, Glendora, CA, USA). The backfill was accomplished with Continuous Wave Obturation system (Kerr Dental, Orange, CA, USA) to achieve a three-dimensional fill and to eliminate possible voids (Figure 3(a)). After obturation was completed, the tooth access was temporized with a cotton pellet and Cavit (3M, Two Harbors, MN, USA). The provisional FPD was recemented with Durelon (Figure 3(b)).

### 2.3. Postoperative Follow-Up

The patient was referred to his prosthodontist for permanent restoration and continuation of care. The patient was recalled for a 6-month follow-up to treatment, and the outcome was excellent. The patient has remained asymptomatic since the completion of NSRCT, and radiographic exam remained free of periapical pathosis.

## 3. Discussion

To identify possible causes of this unusual pink tooth case [1], tooth reduction during tooth preparation [2], heat generation during tooth preparation [3], heat generation during polymerization of the provisional material intraorally [4], and hyperocclusion of provisional FPD were carefully reviewed in the order of treatment sequence.

Tooth reduction during tooth preparation maybe a possible cause, according to other case reports. In short term, Goodacre and Spolnik described that the incidence of endodontic treatment required after tooth preparation ranged from 3% to 23%. FPDs have had higher incidence rates than single crowns. It has been assumed that the higher rates are a result of the greater tooth reduction sometimes required to align multiple teeth [3]. Davis et al.'s research has suggested that 2 mm or more of remaining dentine is critical in protecting the pulp following tooth preparation [4]. In long term, Cheung et al. reported that the pulp survival rate in vital teeth restored with a single-unit ceramic metal crown (CMC) was 84.4% after 10 years. Pulp survival rate of teeth used as abutments for FPD's was 70.8% after 10 years. In addition, maxillary anterior teeth serving as FPD abutments developed pulpal necrosis more frequently than any other tooth type [5]. Figures 1(c) and 1(d) show both teeth #6 and #11 look similar with respect to tooth reduction, and estimated 1.5mm remaining dentin after tooth preparation.

Another possible cause is heat generation during tooth preparation. Historically, Langeland K and Langeland LK obtained histological evidence that crown preparation in human teeth produced no initial pulpal reaction as long as it was carried out with adequate water spray. However, if the water spray was insufficient, the dentine showed evidence of burning, and odontoblast cell bodies were displaced into the ends of the cut dentinal tubules [6]. A temperature rise of 5.6°C can lead to a 15% loss of vitality in the pulp, and an 11°C temperature rise will lead to a vitality loss of approximately 60%. In addition, a 16.6°C temperature rise may cause 100% necrosis of the pulp [7]. In our unique pink tooth case, the dentist who prepared teeth #6 and #11 reported using the same well-maintained handpiece with water throughout the procedure.

Heat generation during fabrication of a provisional FPD using a direct technique could be another factor. Direct and indirect methods are used for the fabrication of a provisional FPD. Driscoll et al. found that the temperature increase produced by the polymerization of dental materials ranged from 14.8 to 27.3°C. A disadvantage of the direct technique is the generation of heat during polymerization of the provisional material intraorally [8]. Due to its accuracy and pulpal protection from polymerization heat, the indirect method is preferred over the direct method; however, time limitation and inadequate laboratory support may require the clinician to use the direct technique [9]. Even though the temporary FPD for this pink tooth case was lab fabricated, it was then relined intraorally with acrylic material, which is a direct technique. It can be speculated that a higher volume of acrylic was used to reline the retainer of tooth #11 than for tooth #6.

It is generally believed that the pink tooth of Mummery is caused by granulation tissue undermining a necrotic area of the coronal pulp. This may be found in asymptomatic teeth with internal root resorption or external cervical resorption [1]. However, in our case, the coronal pulp was vital and there was no clinical evidence of granulation tissue (Figures 2(a)–2(c)). In addition, there was no radiographic evidence of resoptive lesions.

Finally, hyperocclusion of a provisional FPD could be another factor causing pink tooth in this case. It is reported that pink tooth often emerges after a dental trauma or following orthodontic treatment [2]. The patient had sensitivity with percussion pain on tooth #11 (Table 1). Radiographic examination of tooth #11 (Figure 1(d)) was unremarkable. Hyperocclusion may have resulted been a local force exerted on tooth #11. Radiographs of anterior maxilla reveal teeth #6 and #11 and a six unit provisional FPD (Figures 1(c) and 1(d)). Since only two abutment teeth (#6 and #11) were supporting all forces exerted on the six unit provisional FPD (#6, #7, #8, #9, #10, and #11), it is possible that local force was concentrated more on tooth #11 than tooth #6.

In conclusion, the pink tooth of Mummery may occur after a routine tooth preparation in addition to the more traditionally accepted etiology. The tooth discoloration described in this case report may have resulted from tooth preparation [1], heat generation during tooth preparation [2], heat generation during polymerization of the provisional material [3], and/or hyperocclusion of the provisional FPD [4]. By better understanding the multiple etiologies of the pink tooth of Mummery, clinicians may be better able to predict and prevent adverse outcomes of tooth prepration and provisionalization.

## Figures and Tables

**Table 1:** Summary of diagnostic tests.

Tooth	#6 (maxillary right canine)	#11 (maxillary left canine)
Percussion pain	No	Yes
Palpation pain	No	No
Cold test response	Yes	Yes
Mobility	No	No
Swelling	No	No
Probing	<4 mm	<4 mm
Discoloration	No	Yes
